# Hybridization of Surface Plasmon Polariton and Photonic Crystal Modes in Bragg Mirror with Periodically Profiled Metal Film

**DOI:** 10.1186/s11671-016-1357-1

**Published:** 2016-03-15

**Authors:** Mariya V. Sosnova, Sergii V. Mamykin, Alexander V. Korovin, Nicolas L. Dmitruk

**Affiliations:** V.Ye. Lashkaryov Institute of Semiconductor Physics, NAS of Ukraine, 41 prospect Nauki, Kiev, 03028 Ukraine

**Keywords:** Photonic crystals, Surface plasmon polaritons, Bragg mirror, Modes hybridization

## Abstract

The hybridization of the plasmonic and guided modes in the case of one-dimension photonic crystal based on Bragg mirror terminated by a corrugated metal film has been demonstrated theoretically. The simulations have showed that the hybrid plasmonic-photonic mode is characterized by low broadening due to redistribution of the electric field intensity between photonic mode and surface plasmon polariton. It was found that the *Q*-factor and the polarisation sensitivity of these modes are about 144 and 25, respectively, that is 3 times greater than for surface plasmon polariton exciting in similar structure without Bragg mirror.

## Background

Nowadays, the excitation of surface plasmon polariton (SPP) has been widely used in many branches of science and technology, for example, in spectroscopy, photovoltaics [1, 2], sensorics [3], medicine [4] and etc., due to its resonant behavior and ability to confine field in a sub-wavelength volume. It is known, that SPP is a surface wave propagating along interface between two media, when one of them is surface active (with negative permittivity). The main property of SPP is strong localization of electric field with a maximum at the interface [5, 6]. However, one of the significant disadvantage of the SPP is a relatively high dissipation that essentially limits their application.

Today there are several solutions of this problem. On the one hand, it is the search of novel materials with a lower dissipation [7]. On the other hand, it is the utilization of the SPP modes interaction in multi-interface system, for example, the excitation of long-range SPP [8, 9] and channel SPP [10]. The relatively new concept is the design of complex nanomaterials with controlled optical properties. A major focus of these researches is concentrated on the idea of combining the properties of photonic and plasmonic nanostructures [11–13]. As a result, a compromise between dissipation and electric field confinement can be reached.

In this work, the possibility of excitation of hybrid low-dissipative modes in the one-dimensional photonic crystal based on Bragg mirror terminated by a corrugated metal film located on semiconductor substrate has been considered. In this configuration, the metal film might be simultaneously used for the formation of Schottky barrier. Thus, this configuration allows to realize the conversion of the optical signal into electrical one. And this element can be used for connection between the photonic and electronic circuits. In addition, the sensoric properties of structures under consideration have been analysed.

### Mode Hybridization in Plasmonic-Photonic Structures

It is known that SPP is a surface waves propagating at the interface between the metal and the dielectric. Its quality factor can be increased by enhancing the localization of the electric field at this interface [6]. This localization can be improved by using a photonic crystal (PhC) [14] instead of a homogeneous dielectric medium in a regime when the dispersion of SPP is located in the bandgap of the PhC.

**Fig. 1:**
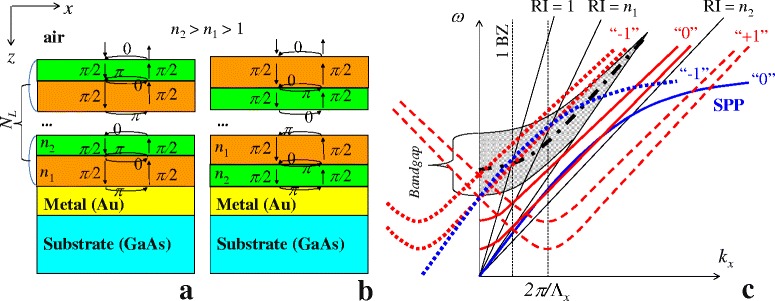
**a**, **b** Schemes of Bragg mirror in the case of different alternating orders of *n*_1_ and *n*_2_ layers. **c** Schematic illustration of modes existing in 1D PhC terminated by a periodically profiled metal film in *p*-polarization for various diffraction orders: 0 (*solid lines*), “ −1” (*dotted lines*) and “ +1” (*dashed lines*). *Blue lines* correspond to the dispersion of SPP, *red lines* – guided modes and *black dash-dotted line* – defect mode. RI = 1, *n*_1_, *n*_2_ mark light lines in media with corresponding refractive indices. *Vertical dotted lines* correspond to first and second Brillouin zones (BZ)

The principal schema of the 1D PhC formed by Bragg mirror confined the metal film on semiconductor substrate is presented in Fig. 1a and b. The Bragg mirror consists of *N*_*L*_-folds sandwich of two alternating dielectric layers with different refractive indices *n*_1_ and *n*_2_ (*n*_2_>*n*_1_>1) with thicknesses (*d*_1_ and *d*_2_, respectively) corresponding to a quarter of the resonance wavelength, *λ*_*BM*_. For this resonant wavelength, the light transmission through Bragg mirror is minimal. In the case of an infinite number of alternating layers (*N*_*L*_→*∞*) forming Bragg mirror, this minimum is transformed into the 1D photonic bandgap (grey region in Fig. 1c) and the resonance wavelength (*λ*_*BM*_) corresponds to the middle of this bandgap. In the case of a finite *N*_*L*_, the coupling of the incident wave with the SPP at the lower boundary of the system 1D PhC terminated by a metal film (“1D PhC-metal film”) can be realized, but quasi-bandgap near *λ*_*BM*_ still remains. Therefore, direct detection of the optical signal of the light transmission into the semiconductor substrate can be carried out using a photodetector based on surface-barrier heterostructure (“Au-GaAs”).

For structure under consideration, there are two systems according to the alternation of layers in Bragg mirror, as shown in Fig. 1a,b. In the first case, when the metal is in contact with an optically less dense layer (*n*_1_) of Bragg mirrors (Fig. 1a), the *π*-phase shift for the round trip of the light at the resonance wavelength appears in each layer of Bragg mirror. In the second case, when the metal is in contact with an optically denser layer (*n*_2_) of Bragg mirrors (Fig. 1b), the *π*-phase shift for round trip of the light at the resonance wavelength appears just for the internal layers of Bragg mirror, whereas the 2*π*-phase appears for two outer layers of Bragg mirror. For this case, there is the defect mode of PhC with dispersion inside bandgap (dash-dotted line in Fig. 1c). This mode is associated with periodicity breakage in the PhC, so it is also called the Tamm polaritons by analogy with surface electron states in solids [15]. Thus, the addition mixing between SPP, PhC and defect modes is appeared. However, the hybridization of the defect mode and SPP is not interesting in the sense of decreasing of SPP dissipation due to large broadening of the defect mode [16].

In the case of a finite number of layer pairs in Bragg mirror, the quasi-bandgap of PhC is formed by weakly localized Fabry-Perot resonances in radiative region which is located between the energy axis and the light line for the *RI*=1 in Fig. 1c (dotted pattern fill). Outside of this region, Fabry-Perot resonances or guided modes (GM) are characterized by stronger spatial localization, but they cannot couple with incoming plane wave (Fig. 1c). For illustrative purposes, there are just two GM dispersions in Fig. 1c (red solid lines) that correspond to a quasi-1D PhC consisting of two pairs of alternating layers. In addition, the SPP dispersion is also presented in Fig. 1c (blue solid line). We can see from Fig. 1c, there is the overlapping between dispersions of GM and SPP in the region between two light lines for media with refractive indices *n*_1_ and *n*_2_. This SPP/GM overlapping leads to the mixing of these modes with hybridization of their properties. In the system with planar translation, all mode dispersions (SPP, GM, and their hybridization) can be shifted into the radiative region. From the technological point of view, the planar translation can be realized by profiling of metal film. Translated dispersions for “ +1” and “ −1” diffraction orders in the case of 1D periodic relief (along *x*-axis) are presented in Fig. 1c by dashed and dotted lines, respectively.

## Methods

The simulations of the light propagation through periodically profiled multilayer structure are based on the curvilinear coordinates transformation method in the framework of the differential formalism [17].

The presence of peculiarities in the transmittance (or reflectance) is associated with mode dispersion occurring in the system. Therefore, to determine the mode dispersions, the transmittance is calculated as a function of photon energy and the planar components of the wave vector of the incident plane wave for two different polarizations of light: transverse electric (*s*-polarization) and transverse magnetic (*p*-polarization) with the direction of the planar periodicity of the metal film profile lying in the plane of incidence. For convenience, the calculated transmittance is represented as a colormap with photon energy along the ordinate, and the planar component of wave-vector along the abscissa. Moreover, both polarizations are represented on the same graph, a planar component of wave vector is counted in a positive direction of the abscissa for *p*-polarization, and in the opposite direction for *s*-polarization. The obtained peculiarities in the transmission for 1D PhC terminated by a periodically corrugated metal film are compared with the mode dispersions for an equivalent stratified planar structure taking into account planar translation [18]. The dispersions of modes in the stratified planar structure were calculated by the transfer matrix method. For the system under consideration with planar periodicity along *x*-axis, mode dispersion can be determined in the following form: $\omega _{mode}(k_{x}) = \omega ^{(0)}_{mode}(k_{x}+m_{x} G_{x})$, where $\omega ^{(0)}_{mode}(k_{x})$ is the mode dispersion for an equivalent stratified planar system, *k*_*x*_ is the planar component of wave vector, *m*_*x*_=0;±1;±2;… is the diffraction order, *G*_*x*_=2*π*/*Λ*_*x*_ is the reciprocal vector for the interface profile with the period *Λ*_*x*_. This method allows to determine mode dispersions in the structure with a planar periodicity with high accuracy except regions of dispersion branches intersection, where mode coupling may be occur. Furthermore, to determine the nature of the modes, the spatial distribution of the electric field intensity in the plane of incidence is used in the work.

To clarify resonance properties, we use quality factor (*Q*-factor) in the form *Q*=*λ*_*res*_/*Δλ*, where *λ*_*res*_ is the resonant wavelength and *Δλ* is the width at half height of resonant peak. To analyse polarisation sensitivity, we use ration between transmittance for *p*- and *s*-polarisation of the light (*T*_*p*_/*T*_*s*_).

## Results and Discussion

**Fig. 2:**
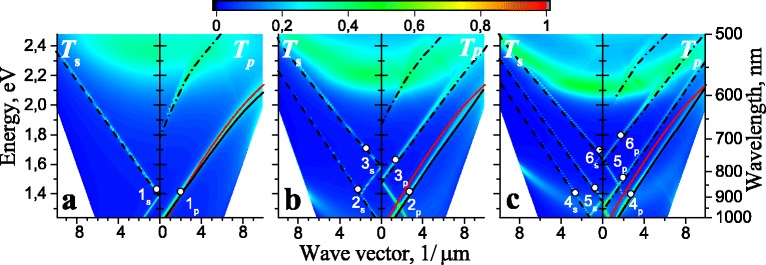
Calculated transmittance of *s*- and *p*- polarized light as function of in-plane component of the wave vector and photon energy through Bragg mirror on sinusoidal profiled gold film with a thickness of 40 nm into GaAs substrate for various numbers of layer pairs (*n*_1_ and *n*_2_) in Bragg mirror: **a** one **b** two, and **c** three. The profiling period of metal film is 700 nm and depth is 50 nm. The *lines* correspond to “ −1” diffraction order for *s*- (*dashed lines*) and *p*- (*dash-dotted lines*) guided modes and SPP (*solid lines*). *Red lines* correspond to the dispersion of SPP for “ −1” diffraction order in the case of flat interface between gold and uniform dielectric with refractive index *n*_1_

In this section, we consider optical response of plasmonic-photonic structure: 1D PhC on surface-barrier heterostructure Au/GaAs with periodical-profiled metal film (“1D PhC/sinusoidal profiled Au/GaAs”) in order to identify the resonances with large *Q*-factor and peak intensity for use in sensorics. The dependence of light transmittance through structure “1D PhC/sinusoidal profiled Au/GaAs” as function of in-plane component of the wave vector and photon energy is presented in Fig. 2 for both polarisations of light with the direction of periodicity lying in the plane of incidence. The structure parameter were chosen in the way that allows to observe the formation of 1D photonic bandgap in an energy range of 1.4÷2.2 eV. In the simulations for Bragg mirror, we used *λ*_*BM*_=700 nm, *n*_1_=1.47, and *n*_2_=2, whereas the number of layer pairs, *N*_*L*_, is varied from 1 to 3. We chose this number of layer pairs to facilitate the analysis, namely, to decrease the number of analyzed modes, since the number of layer pairs directly determines the number of modes. And, to trace the modifications of mode dispersions by increasing the amount of layer pairs in the Bragg mirror. A gold film with a thickness of 40 nm is sinusoidally profiled with a period of 700 nm and a profiling depth of 50 nm. The optical constants of gold and GaAs were taken from [19] and [20], respectively.

The mode dispersions for equivalent planar multilayer structure are added in Fig. 2 taking into account dispersion shift due to planar periodicity. Dispersions of *s*- and *p*- guided modes are marked by dashed and dash-dotted lines, respectively. The dispersion of SPP modes is marked by solid lines. Such dispersions for “ −1” diffraction order coincide with transmittance peculiarities in Fig. 2. In the case of *N*_*L*_-folds, Bragg mirror there *N*_*L*_ modes in *s*-polarisation and (*N*_*L*_+1) modes in *p*-polarisation. The lower dispersion curve in *p*-polarisation corresponds to SPP and it redshifts with increasing of layers in Bragg mirror, *N*_*L*_. The dispersion of “conventional” SPP in the case of flat interface between gold and dielectric with refractive index *n*_1_ is added too (red lines in Fig. 2). We should note that dispersion of lower *p*-mode (black solid lines) is more linear than the “conventional” one-interface SPP that may be associated with SPP hybridization. Also, we can see from Fig. 2 that *p*-polarized GM are blueshifted in comparison with *s*-polarized GM.

**Fig. 3:**
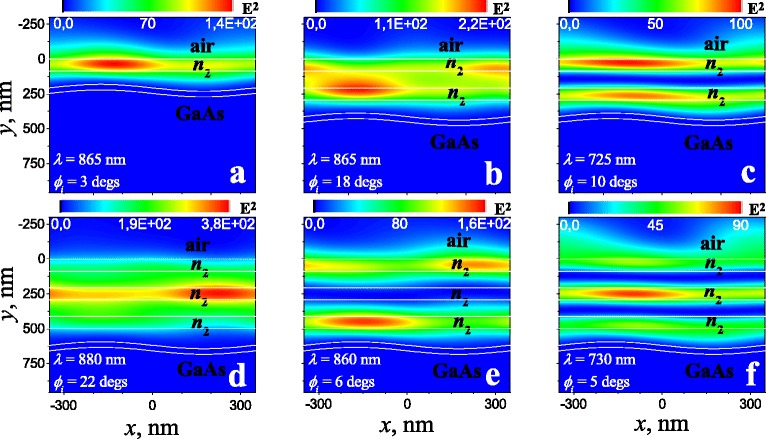
Spatial distribution of electric field intensity (*E* ^2^) in *s*-polarisation for “1D PhC/sinusoidal profiled Au/GaAs” for various numbers of layer pairs in Bragg mirror at the points indicated in Fig. 2: **a** 1 _*s*_, **b** 2 _*s*_, **c** 3 _*s*_, **d** 4 _*s*_, **e** 5 _*s*_, and **f** 6 _*s*_. Light is incident on the *top* of structure. Other parameters are the same as in Fig. 2

**Fig. 4:**
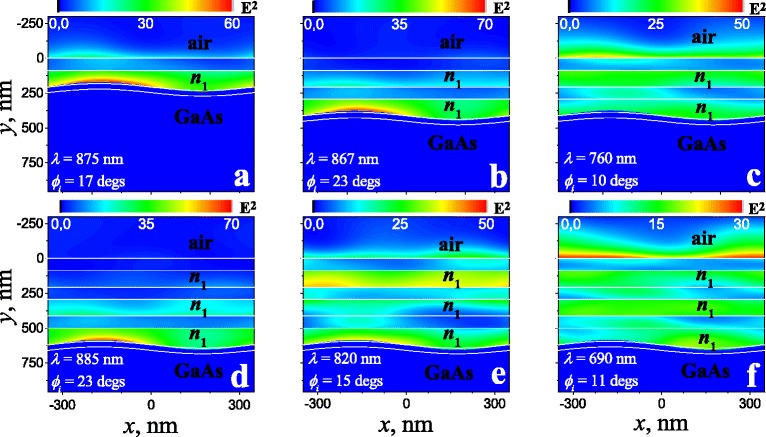
Spatial distribution of electric field intensity (*E* ^2^) in *p*-polarisation for “1D PhC/sinusoidal profiled Au/GaAs” for various numbers of layer pairs in Bragg mirror at the points indicated in Fig. 2: **a** 1 _*p*_, **b** 2 _*p*_, **c** 3 _*p*_, **d** 4 _*p*_, **e** 5 _*p*_, and **f** 6 _*p*_. Light is incident on the top of structure. Other parameters are the same as in Fig. 2

To analyse the origin of modes, the spatial distribution of the electric field intensity for dispersion points marked by circles in Fig. 2 is presented in the Fig. 3 for *s*-polarization and Fig. 4 for *p*-polarization in the case of various numbers of layer pairs forming Bragg mirror. Specific points were chosen to trace the changes in the spatial distribution of the electric field for all modes (except the topmost *p*-polarized mode) outside of their intersection and for “-1” diffraction order. There is no localization of the electric field near the interface with the metal for the topmost *p*-polarized mode in contrast to other *p*-polarized modes, so the spatial distribution of the electric field is not shown in Fig. 4 for this mode. As we can see from Fig. 3, the spatial localisation of the electric field for *s*-polarized GMs is situated in more optical dense layers of Bragg mirror (*n*_2_-layers). In general, the nature of electric field localization for *s*-polarized modes corresponds to the spatial distribution of the wave function in multiple-quantum-wells. Thus, the transverse spatial distribution of the electric field intensity for *N*_*L*_-fold Bragg mirror can be estimated in the framework of multiple wells theory [21] in the form of superposition: $I(z)=\Sigma _{n=1}^{N_{L}}c_{n} I_{n}(z)$, where *I*_*n*_(*z*) is the transverse spatial distribution of the electric field intensity for fundamental modes in each *n*^th^ well formed by *n*_2_-layer of the Bragg mirror. Energy for such coupled modes and their amplitudes are determined by the eigenvalue problem in the form

(1) $$ \left[\left(\begin{array}{cccccc} 0 & T & & 0\\ T & \ddots & \ddots & \\ & \ddots & & T\\ 0 & & T & 0 \\ \end{array}\right)-\omega \right] \left(\begin{array}{c} c_{1} \\ c_{2} \\ \dots \\ c_{N_{L}} \\ \end{array}\right)=0,  $$

where *ω* is the mode frequency, *T* is the tunnel matrix element defined by the overlapping of electric field intensity of neighbour modes. Following to Eq. 1, there are two *s*-modes in the case of twofold Bragg mirror with dispersion *ω*^(1,2)^(*k*)=*ω*_0_(*k*)±*T* with amplitudes: $c^{(1)}=\left \{1;1\right \}/\sqrt {2}$ and $c^{(2)}=\left \{1;-1\right \}/\sqrt {2}$, where *ω*_0_(*k*) is the dispersion of fundamental mode in the case of “ *n*_1_/*n*_2_/*n*_1_” structure with spatial distribution similar to one presented in Fig. 3a. Such amplitudes define symmetric and asymmetric distributions of electric field intensity that can be observed in Fig. 3b, c, respectively. By analogy, there are three *s*-modes in the case of threefold Bragg mirror with dispersion: $\omega ^{(1,3)}(k)=\omega _{0}(k)\pm \sqrt {2}T$ and *ω*^(2)^(*k*)=*ω*_0_(*k*) with amplitudes: $c^{(1)} = \left \{1;\sqrt {2};1\right \}/2$, $c^{(2)}=\left \{1;0;-1\right \}/\sqrt {2}$ and $c^{(3)} = \left \{1;-\sqrt {2};1\right \}/2$. For them, the spatial distribution of electric field intensity can be observed in Fig. 3d for *ω*^(1)^(*k*), Fig. 3e for *ω*^(2)^(*k*), and Fig. 3f for *ω*^(3)^(*k*).

In opposition to *s*-polarized modes, the highest electric field intensity for *p*-modes is located in the less optical dense media (*n*_1_-layers) and near to the metal interface. And the electric field intensity is redistributed between Bragg mirror layers with the refractive index *n*_1_ and metal interface depending on the dispersion branch (Fig. 2). Thus, the electric field intensity is maximal on the metal interface with Bragg mirror for the lower mode (Fig. 4a, b and d), whereas the electric field intensity is maximal at the boundary of Bragg mirror with the environment for the second (Fig. 4c) and third (Fig. 4f) modes. But in any case there is local or global maximum of the electric field intensity at the metal interface with Bragg mirror for *p*-modes. This fact is an argument for hybrid plasmonic-photonic nature for *p*-polarised modes.

**Fig. 5:**
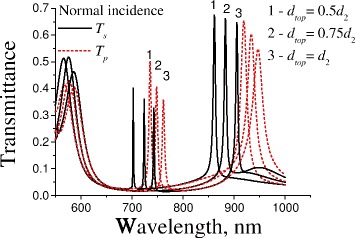
Calculated transmittance spectra for *s*- and *p*-polarized light through the multilayered plasmonic-photonic structure “1D PhC/sinusoidal profiled Au/GaAs” for various thicknesses of the front layer (*d*_*top*_). The number of layer pairs in Bragg mirror is three. Other parameters are the same as in Fig. 2

**Fig. 6:**
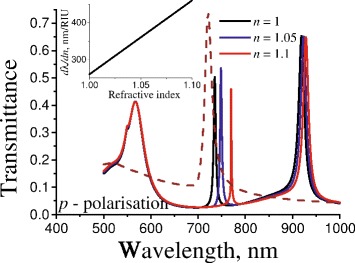
Calculated spectra of transmittance for *p*-polarized light through the multilayered plasmonic-photonic structure “1D PhC/sinusoidal profiled Au/GaAs” for various refractive index of the environment. *Dashed line* corresponds to the transmittance of similar structure without Bragg mirror. *Inset* presents the spectral sensitivity on variation of the refractive index of environment. Other parameters are the same as in Fig. 5 for *d*_*top*_=*d*_2_

The spectral position of guided and hybrid modes strongly depends on the dielectric layer thicknesses. The calculated spectra of transmittance for both polarizations of normally incident light (the direction of periodicity is lied in the plane of incidence) through “1D PhC/sinusoidal profiled Au/GaAs” for varied thickness of front layer in the Bragg mirror, *d*_*top*_, is presented in Fig. 5. In these simulations, we used quasi 1D PhC consisting of threefold Bragg mirror. As we can see from Fig. 5, there is quasi-bandgap in a range of 600÷900 nm (1.38÷2.07 eV) with transmittance below 5 %. Inside this bandgap the extremely narrow resonances are observed for both polarizations of light. The *Q*-factor for *p*-polarised mode at *λ*≃740 nm is equal to 144. The polarisation sensitivity of this mode is 25 for *d*_*top*_=*d*_2_ and decreases with decreasing of *d*_*top*_. For similar structure without Bragg mirror, the *Q*-factor is about 46 with a polarisation sensitivity of 8.6 (its transmittance spectrum for *p*-polarised light is presented in Fig. 6 by dashed line). So, the presence of Bragg mirror increases the *Q*-factor and the polarisation sensitivity three times.

In addition, the spectral position of guided and hybrid modes is sensitive to changes of refractive index of the environment. The calculated spectra of transmittance of normally incident *p*-polarized light through multilayered plasmonic-photonic structure “1D PhC/sinusoidal profiled Au/GaAs” under consideration for the varied refractive index of the environment is presented in Fig. 6. The transmittance spectra of the same structure without Bragg mirror (“sinusoidal profiled Au/GaAs”) is added in Fig. 6 to compare properties of SPP and hybrid modes. The spectral sensitivity on the variation of environment refractive index is presented in the inset of Fig. 6. As we can see from Fig. 6, the “1D PhC/sinusoidal profiled Au/GaAs” structure demonstrates a sensitivity of about 400 nm/RIU that is two times less than for the same structure without Bragg mirror. But there is an essential increase in the slope of the resonance. Namely, the maximum value of derivative *∂T*/*∂λ* riches 0.35 nm ^−1^ in the case of structure of the consideration, whereas this value is equal to 0.053 nm ^−1^ in the case of similar structure without Bragg mirror. This means that such narrow resonances possess higher sensitivity on the signal intensity changes in the case of a weak variation of the refractive index of the environment.

## Conclusions

In the framework of differential formalism, the possibility of the excitation of hybrid plasmonic-photonic modes in multilayered structures with quasi 1D photonic crystal based on Bragg mirror terminated by a periodically-profiled metal film in a spectral region close to the visible has been analyzed theoretically using a curvilinear coordinate transformation method. The metal film permittivity was assumed to be uniform with permittivity close to bulk one. As shown by numerical simulations, the hybrid mode for *p*-polarized light is characterized by low broadening due to the redistribution of the electric field intensity between the metal interface and Bragg mirror with concentration of electric field in the optically less dense layers of Bragg mirror. Calculations showed that the quality factor of such mixed SPP-photonic modes in *p*-polarization is about 144 which is three times greater than for SPPs in similar structure without Bragg mirror. Furthermore, the polarization sensitivity of SPP is significantly increased from 8.9 to 25 by adding Bragg mirror on the surface of the profiled metal film due to the appearance of the photonic quasi-bandgap. This complex plasmonic-photonic structure is a promising element for construction of highly sensitive selective active sensor and polarization filters. Also, such plasmonic-photonic structures can be useful to improve the surface-enhanced Raman scattering, optoelectronic devices, and plasmonic circuits.
